# Keystroke Dynamics-Based Authentication Using Unique Keypad

**DOI:** 10.3390/s21062242

**Published:** 2021-03-23

**Authors:** Maro Choi, Shincheol Lee, Minjae Jo, Ji Sun Shin

**Affiliations:** 1 Department of Computer and Information Security, Sejong University, Seoul 05006, Korea; qkqnfld@sju.ac.kr (M.C.); eklim2@sju.ac.kr (M.J.); 2 Department of Computer and Information Security, and Convergence Engineering for Intelligent Drone, Sejong University, Seoul 05006, Korea; sclee@sju.ac.kr

**Keywords:** authentication, keystroke dynamics, machine learning, smartphone, unique keypad

## Abstract

Authentication methods using personal identification number (PIN) and unlock patterns are widely used in smartphone user authentication. However, these authentication methods are vulnerable to shoulder-surfing attacks, and PIN authentication, in particular, is poor in terms of security because PINs are short in length with just four to six digits. A wide range of research is currently underway to examine various biometric authentication methods, for example, using the user’s face, fingerprint, or iris information. However, such authentication methods provide PIN-based authentication as a type of backup authentication to prepare for when the maximum set number of authentication failures is exceeded during the authentication process such that the security of biometric authentication equates to the security of PIN-based authentication. In order to overcome this limitation, research has been conducted on keystroke dynamics-based authentication, where users are classified by analyzing their typing patterns while they are entering their PIN. As a result, a wide range of methods for improving the ability to distinguish the normal user from abnormal ones have been proposed, using the typing patterns captured during the user’s PIN input. In this paper, we propose unique keypads that are assigned to and used by only normal users of smartphones to improve the user classification performance capabilities of existing keypads. The proposed keypads are formed by randomly generated numbers based on the Mersenne Twister algorithm. In an attempt to demonstrate the superior classification performance of the proposed unique keypad compared to existing keypads, all tests except for the keypad type were conducted under the same conditions in earlier work, including collection-related features and feature selection methods. Our experimental results show that when the filtering rates are 10%, 20%, 30%, 40%, and 50%, the corresponding equal error rates (EERs) for the proposed keypads are improved by 4.15%, 3.11%, 2.77%, 3.37% and 3.53% on average compared to the classification performance outcomes in earlier work.

## 1. Introduction

Smartphones not only play an important role as IoT devices in our everyday life, but also will do as crowdsensing entities for the future of smart cities. The more smartphones contribute to, the more they need security and privacy protection. As security solutions for smartphones, authentication methods that use a personal identification number (PIN) and unlock patterns are widely employed for smartphone user authentication. However, these authentication methods are vulnerable to shoulder-surfing attacks, and PIN authentication, in particular, is poor in terms of security because PINs are short in length with just four to six digits. As a way to overcome this limitation, various biometric recognition methods have been developed which are capable of authenticating a normal user based on their unique biometric information. Biometric recognition technology refers to methods that allow the smartphone to learn data that is unique to its user, such as the user’s face [1], fingerprint [2], or iris [3] information. However, if the user fails to enter his or her biometric recognition information properly, or exceeds the set maximum number of authentication attempts, PIN-based authentication is then required as the final authentication step. If, by any chance, an intruder knows the user’s PIN, the smartphone in question will permit the intruder access during the PIN input stage.

To prevent such attacks by intruders, research has been conducted on keystroke dynamics-based authentication, where users are classified by analyzing their typing patterns as they enter their PIN. Furthermore, in an effort to improve the user classification performance of these methods, there have been various studies conducted on diversifying the features that can be extracted from typing patterns, by applying artificial rhythms to facilitate the user classification, or employing a suitable classifier for binary classification.

Unlike existing methods, the method proposed in the present study was designed to improve user classification performance by assigning a unique keypad to the normal user during the user classification process. Using the feature selection algorithm presented in [4], the data collected from the unique keypad was subject to feature selection and then preprocessing, followed by user classification tests using three types of classifiers: Linear support vector machine (SVM), One-Class SVM, and Manhattan distance-based classifiers. The test results obtained using the unique keypad were then compared with those obtained using a conventional keypad. The results confirmed that the user classification performance was better in both analytical and practical aspects when the proposed unique keypad was employed.

The organization: In Section 2, previous studies on keystroke dynamics-based authentication and keystroke dynamics-based user classification using keypads are reviewed. In Section 3, feature data collection and extraction-related features, which are employed for improved classification performance, are described. Section 4 reviews the development process and principles of the designed unique keypad. In Section 5, test results obtained from the proposed unique keypad are presented, and these results are further compared and analyzed in terms of performance. Finally, in Section 6, the major experimental findings are discussed along with future research directions.

## 2. Related Work

In this section, previous studies on keystroke dynamics-based authentication, as well as the papers that have been published on user classification for authentication, are discussed.

### 2.1. Keystroke Dynamics-Based Authentication

Keystroke dynamics-based authentication is a common behavioral biometric approach, in which users are identified based on their typing patterns and rhythms, which are collected as they use PC keyboards or smartphone touchscreens. The concept of keystroke dynamics, which is a user authentication method based on behavioral patterns, i.e., their keyboard typing patterns, first appeared in 1975 [5]. Based on this keystroke concept, it was demonstrated in 1985 that a false rejection rate (FRR) of 12% and a false acceptance rate (FAR) of 6% could be achieved in classification tests [6]. The authentication tests used the difference in time between each keystroke, which was measured and extracted from the subjects’ inputs in the keyboard.

In 2009, a study verified that the number of training data and how the collected data were normalized were very important for improving the keystroke data-based user classification performance [7]. 30 test subjects inputted 10 characters 20 times each, and these data were then subjected to both user-dependent score normalization and user-independent score normalization methods. Each classification test was conducted while varying the number of training data (5 to 10 data points out of the 20 collected data for each subject). It was found that the results were best when the number of training data sets for each subject was set to 10, with an equal error rate (EER) of 14.46% and 16.21%, respectively. This keystroke dynamics concept has been used for user authentication applications not only in a PC environment but also in a mobile environment. In a smartphone-based authentication study in 2002, keystroke dynamics were employed to classify four-digit PINs and fixed 11-digit mobile phone numbers [8]. The average time taken to complete the set key inputs, the standard deviation of the time, and the delay time between each keystroke were used as data features. As a result, the EER was calculated to be 15% for the four-digit PINs and 15% for the fixed 11-digit mobile phone numbers.

In a 2018 study, with more advanced smartphone technology, there was an attempt to classify users using the data measured by a motion sensor built into a smartphone [9]. These results were compared with the existing results obtained without using motion data. An acceleration sensor, angular velocity sensor, and rotation-vector sensor were used as the motion sensors. The mean, root mean square, sum of positive numbers, sum of negative numbers, and standard deviation of the data measured by each sensor were calculated, and suitable features for classification were selected accordingly. The EER for existing user classification results obtained without motion sensor data was measured to be 8.94%. That figure decreased by 1.05% to 7.89% when the mean feature was applied, among the motion sensor-based features. The mean feature was the best performance among the motion sensor-based features. These results demonstrated that it was possible to improve user classification performance by employing feature data extracted from motion sensor data.

Recently, several studies have applied keystroke dynamics-based authentication in various environments. These include studies based on keystroke dynamics from free text, in contrast to other work that utilized fixed length elements, such as passwords or PINs [10,11], as well as research that continuously classifies the user’s keystroke dynamics [11,12,13] and studies that consider various typing positions, such as sitting, walking, and relaxing positions [14].

In addition to keystroke dynamics-based authentication, user authentication research based on various biometric factors has been very active. Biometric authentication methods based on face [1], fingerprint [2], and iris [3] information are widely used, and recently, authentication methods based on information such as that generated by electroencephalogram (EEG) [15] and electrocardiogram (ECG) [16] are attracting attention as part of the effort to resolve the vulnerabilities of existing biometric authentication approaches, such as the forgery of biometric information.

#### 2.1.1. Random Keypad

As smartphones become more embedded in our daily lives, sensitive user information (such as bank account passwords, smartphone PINs, and text messages) are being increasingly stored in them. In an attempt to secure and protect such sensitive information from various attacks, research has been conducted on the PIN input method using random keypads, rather than existing conventional keypads. In a 2010 study, the time taken to complete four-digit and eight-digit numbers was measured using a random keypad in a mobile environment [17]. The number of keystroke errors was also measured, and all these results were compared with those obtained using a normal keypad. In the case of four-digit numbers, when using the random keypad, the keystroke completion time was over 1 s longer, but the error rate was 0.01% lower, showing improved performance, compared to using the normal keypad. In the case of eight-digit numbers, the keystroke completion time was over 3 s longer, and the error rate was 0.03% higher. These results verified the usability of a random keypad for short PINs, such as four-digit ones.

Various other methods were proposed to improve the vulnerability of using a normal keypad, which are vulnerable to side-channel keystroke inference attacks, for example [18]. These included changing the arrangement and size of the keypad buttons. In the study, effective protective measures against side-channel keystroke inference attacks were proposed, as follows: row randomization (RR) where the position of the numbers in the four rows of the 4 × 3 keypad is changed, column randomization (CR) where the position of the numbers in the three columns is changed, individual key randomization (IKR) where the position of the keypad buttons is randomly changed, gray-scale IKR where each button of the IKR keypad is colored differently, key size randomization (KLR) where the size of the keypad buttons is changed, and keypad location randomization (KLR) where the keypad is divided into a 7 × 6 grid, and some of these grid points are selected and arranged to form a 4 × 3 keypad.

In a 2019 study, the test subjects were asked to input fixed PINs using a random keypad that was designed to change every round [19]. User classification performance was measured and analyzed based on the collected data. More specifically, the 30 test subjects entered fixed 10-digit PINs using a random keypad set for ten rounds. Based on the entered data, 32 features (including the time taken to press and release a single button, the time between consecutively inputted keys, the range of area touched by the fingertips when pressing each button, and the gender) were collected, and a random forest classifier was applied. As a result, the EER was measured to be 10%.

Unlike our study, which provides a keypad that collects unique features that represent only normal users, the aforementioned studies based on random keypad techniques [17,19] cannot readily collect data pertaining to unique features well enough to classify normal users effectively, as they provide keypads that are not familiar to everyone, including normal users. In addition, in one study [18], the authors focused on evaluating the usability of randomized keypads to prevent keystroke inference attacks by malicious attackers. Here, on the other hand, we focus on verifying how normal users are better classified relative to abnormal users through the provision of unique keypads.

#### 2.1.2. Feature Selection

There was a study on feature selection for keystroke data, which can be used to classify normal and abnormal users in a smartphone environment. In a 2003 study, 21 participants were asked to enter the four-digit key “abcd”, and, as a result, time vector data were obtained, which were composed of the duration time and interval time [20]. The data were then subjected to a genetic algorithm, and based on this result, feature selection methods were examined to improve the classification performance. In a 2007 study, time vector data composed of duration time and interval time was collected while 24 participants entered the four-digit key “abcd”, and was subjected to PSO (particle swarm optimization) [21]. Feature selection methods were examined in an attempt to improve the classification performance.

In 2009, data were collected from 27 test subjects who had different passwords from each other [22]. Based on the collected data, the duration time, latency time, and digraph were extracted as data features, and the mean and standard deviations of the extracted data were determined. Using the measured values, they attempted to apply the ant colony optimization (ACO) as a way to improve user classification performance. In 2010, based on the data collected from 27 participants, the duration time, latency time, and digraph were set as data features, while various feature selection methods, including particle swarm optimization, genetic algorithm, and ant colony optimization, were applied to measure and compare the classification performance [23]. The respective accuracies were measured to be 88.9%, 86.6%, and 92.8%, while the EER was 0.063%, 0.078%, and 0.059%, respectively.

In 2011, an attempt was made to classify users by various methods, including the most frequently typed n-graph selection, quickly-typed n-graph selection, time-stability typed n-graph selection, and time-variant typed n-graph selection [24]. The results showed that the user classification performance was the best when the most frequently typed n-graph selection method was applied. In a 2020 study, based on the trimmed mean and variation coefficients of the collected data, feature scores were determined and compared [4]. Features with low scores were discarded to improve classification performance.

## 3. Keystroke Dynamics

Keystroke dynamics refers to the typing patterns or action types that are generated when a user entering keys on a PC or smartphone. The method of classifying users based on the unique typing patterns generated by each user is called keystroke dynamics-based authentication. In a smartphone environment, a wide range of data can be extracted during the authentication process, as the user repeatedly presses and releases the smartphone screen. Data can include the time interval between each keystroke and the next one, the time taken to press and release a single key, and the movement of the smartphone while the keys are being pressed. Keystroke dynamics-based authentication can be enabled based on these collected keystroke data. Keystroke data can be largely divided into touch data and motion data, and these are extracted according to various criteria.

### 3.1. Touch Data

Touch data are generally recorded at the moment the user touches the smartphone screen (keyDown) and then removes the finger from the screen (keyUp). Three collection features are involved here: the time between each touch on the smartphone screen, the pressure applied by the finger when touching the screen, and the position of the finger both when pressing and releasing the smartphone key.

#### 3.1.1. Time

**Figure 1:**
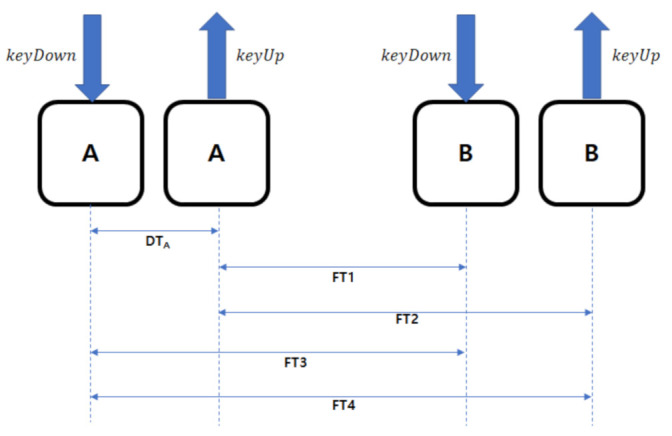
Time feature structure.

- DT (dwell time)
  The DT refers to the time difference between the keyUp and keyDown when entering a single key, as shown in Figure 1. Based on the collected keyDown and keyUp data, the DT can be calculated using Equation (1).
  (1) $$A.keyUp-A.keyDown=DT_{A}$$
- FT (flight time)
  The FT is a feature extracted from the keyDown and keyUp data when two keys are entered. Figure 1 shows four FT features, while Equation (2) is an equation that calculates the FT for each feature, which is the target data to be extracted.
  (2) $$\begin{array}{cc} FT1= & B.keyDown-A.keyUp\\ FT2= & B.keyUp-A.keyUp\\ FT3= & B.keyDown-A.keyDown\\ FT4= & B.keyUp-A.keyDown \end{array}$$

#### 3.1.2. Pressure

Pressure data are recorded by measuring the finger pressure when the user touches the screen and removes the finger from the screen. The data corresponding to when the user is touching the screen with a finger are the downPressure, while the data corresponding to when the finger is removed from the screen are the upPressure.

#### 3.1.3. Coordinate

Coordinate data is recorded by measuring the x and y coordinates of each touch on the screen when the user presses the screen and removes the finger from the screen. The features collected when the user presses the screen are the downX and downY, while those collected when the user removes the finger from the screen are the upX and upY.

### 3.2. Motion Data

**Figure 2:**
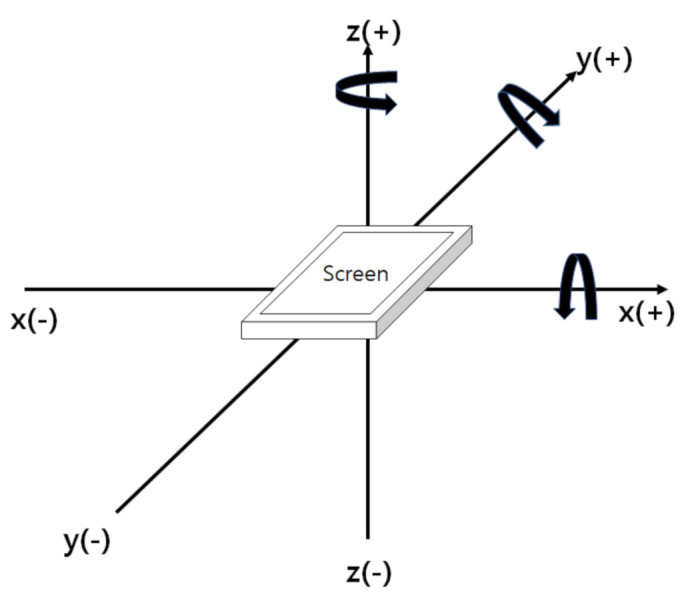
Smartphone reference axis for motion data.

Motion data are collected by measuring the movement of the smartphone while the user is entering the keys on the screen. The motion data are measured based on the three-dimensional coordinates of the *x*-, *y*-, and *z*-axes. As shown in Figure 2, the *x*-axis represents the left and right movement of the smartphone, while the *y*- and *z*-axes represent the up and down movement and the back and forth movement of the smartphone, respectively. The motion data are divided into acceleration, angular velocity, and rotation vectors.

#### 3.2.1. Accelerometer

An acceleration sensor is mainly used to determine the inclination of an object or the degree of vibration, and the values measured by this sensor basically include the effect of gravitational acceleration. The acceleration is measured using this characteristic. Given that the smartphone is affected by gravity even while it is not in use, the acceleration needs to be extracted in the form of linear acceleration by removing the effect of gravitational acceleration. Here, acceleration is indicated as the accelerometer (acc), while the linear acceleration is referred to as the linear accelerometer (lacc).

#### 3.2.2. Angular Velocity

The angular velocity sensor is used to measure the angle at which an object rotates per hour around a specific axis. The angular velocity can be measured using this characteristic. Here, the angular velocity is expressed as the gyroscope (gyr).

#### 3.2.3. Rotation Vector

The rotation vectors refer to the angle at which the smartphone has been rotated relative to each axis. The orientation of the smartphone is measured using a geomagnetic sensor. With a geomagnetic sensor, the measured rotation vectors are affected by the north pole. In order to eliminate the effect of the north pole, rotation vectors are estimated as game rotation vectors, instead of using the values collected by the geomagnetic sensor. The data collected from the accelerometer and gyroscope are used, just as in determining the acceleration and angular velocity. The rotation vectors are indicated as the rotation (rot), while the game rotation vectors are expressed as the game rotation (grot).

## 4. Unique Keypad

In keystroke dynamics-based authentication in a smartphone environment, users are classified by analyzing the PIN input data of the normal user, training the system with the analyzed data, and then allowing the system to classify users who attempt authentication. Here, we denote a legitimate user as a normal user and imposters as abnormal users. This user classification process requires the input data to be unique and consistent. In this regard, the collected data must include the unique feature data that exclusively represent the normal user and the feature data that consistently occur only for the normal user. The features obtained based on these principles are called uniqueness and consistency.

In most previous studies on keystroke dynamics-based authentication, conventional keypads which were familiar to every user were employed. Given that both normal and abnormal users used the same keypad, the features obtained from the collected data failed to exhibit the uniqueness and consistency of just the normal user. For that reason, in other studies investigating ways to improve exiting keypad-based user classification performance, various measures have been proposed, such as a feature selection method suitable for the user [5], a method of improving the quality of collected data using artificial rhythmic cues [25], and a suitable classification method for the features extracted from a mobile environment [26]. However, these papers were involved several problems, for example, there was difficulty finding the optimal number of features to classify a normal user, errors occurred when using complex rhythmic cues, and the use of unoptimized classifiers. In an attempt to overcome these limitations, in the present study, a unique keypad is proposed to extract data that identifies the uniqueness and consistency of the normal user only.

### 4.1. The Proposed Unique Keypads

We employed the Mersenne twister [27] to assign a unique keypad to each user, in which 10-digit random numbers were created while using each number from 0–9 only once, and then these random numbers were accordingly assigned to the keypad. The normal users for each smartphone were asked to enter their PINs using a unique keypad assigned to them. Each smartphone was trained with the input data and then to try and classify users.

Unlike the data obtained using a conventional keypad, the data obtained in the present study were found to be unique and consistent because each normal user was assigned a unique keypad that was familiar to them but not to the others. Thus, it was possible to extract the feature data that were unique to the normal user, and yet were also consistent. Even if other users attempted to enter the same PIN, the user classification performance would still be better.

#### 4.1.1. Uniqueness and Consistency

A keystroke dynamics-based authentication method which collects data using a conventional keypad is also capable of extracting consistent data from the collected user data. However, this conventional keypad is also familiar to users other than the normal user, and thus the data extracted from them may be quite similar to those extracted from the normal user. By using a unique keypad that is assigned to and used by only the normal user, more unique and consistent data of the normal user can be collected, as compared to collecting data using a conventional keypad. Additionally, even if users other than the normal user happen to know the correct PIN, they will be asked to enter the PIN using a unique keypad that is not familiar to them. Therefore, there will be an obvious difference in the data collected from the normal user and any others. By training the smartphone using data collected from the normal user with this method, it is possible to improve its ability to classify abnormal users.

#### 4.1.2. Keypad Configuration

**Figure 3:**
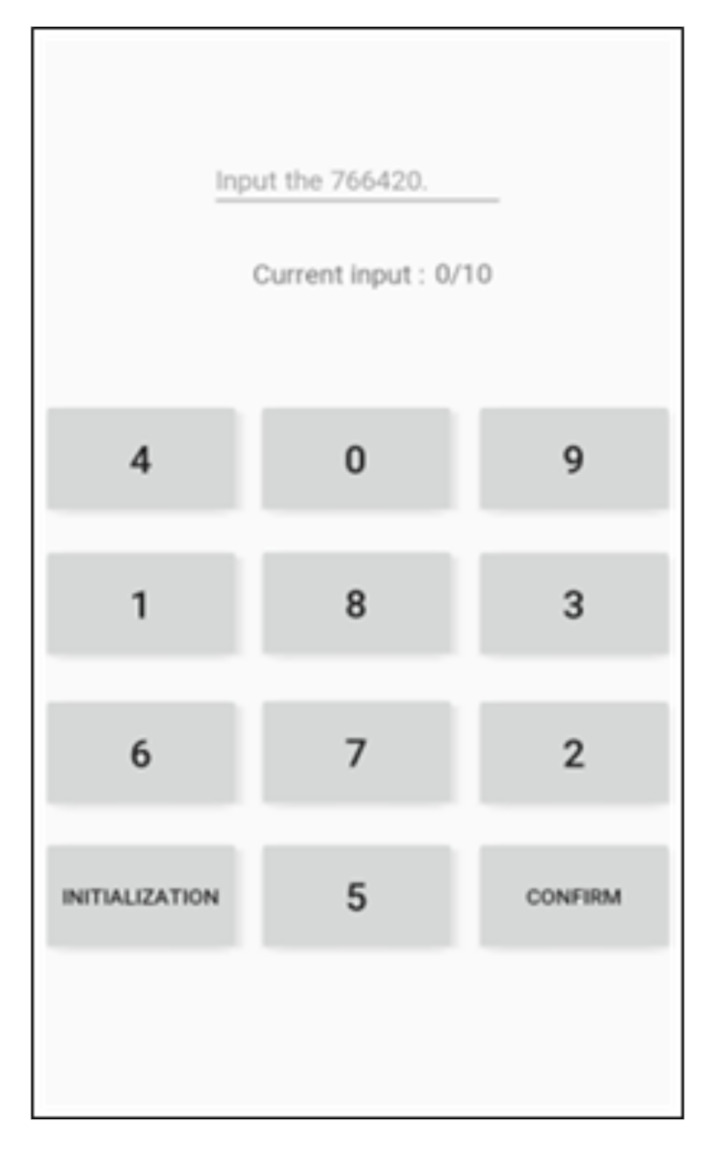
Initial keypad.

**Figure 4:**
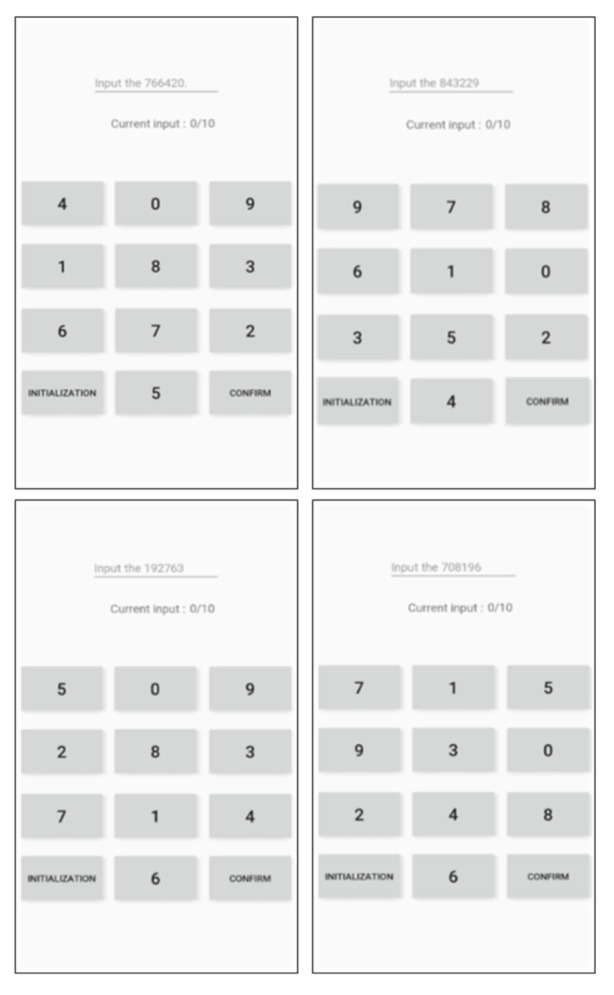
Unique keypad.

We employed the Mersenne twister [27] to assign a unique keypad to each user. We generated 10-digit random numbers while using each number from 0 to 9 only once. Following that, these random numbers were accordingly assigned to the keypad. The seed value used for the random number generation was set to the time when the corresponding unique keypad was generated. Each of the newly generated random numbers was assigned one by one to each of the keypad buttons, except for the INITIALIZATION and CONFIRM buttons, from the first row to the last one and from left to right in each row. The newly generated keypad was assigned to the corresponding smartphone, and the user was asked to enter the PIN using the keypad. While doing so, the user becomes accustomed to his or her own typing patterns. This process allows the smartphone to learn from the input data collected from the normal user. If random users enter the PIN using the same keypad, the smartphone can tell them from the normal based on the collected data. Figure 3 shows the initial keypad arrangement devised to generate a unique keypad, and Figure 4 shows the four keypads used for the tests in the present study.

## 5. Experimental Methods and Results

Tests were conducted to compare the data collected using the proposed unique keypad with those obtained using a conventional keypad, to demonstrate that user classification performance could be improved using the proposed method. The tests were performed as follows. First, data features used for user classification were selected based on the collected data. The selected data features were normalized, and the user classification results were confirmed through classifiers.

This section describes the data collection process for the tests, how data features were selected, data normalization, details about classifiers, and the test results.

### 5.1. Experimental Method

#### 5.1.1. Data Collection and Extraction

Data collection was conducted using the unique keypad and four Nexus 5X devices, which were assigned different six-digit PINs (“766420”, “843229”, “192763”, and “708196”). A total of 13 participants were asked to use one smartphone. The number of participants is a sufficient number, verified as 13 participants in one study [28] and 10 participants in another [29]. Among them, one person was designated as the normal user, while the other 12 were abnormal users. As mentioned in Section 4.1.1, as the unique keypad was assigned to and used by one normal user, in an experiment with one unique keypad, only one user was a normal user. The remaining 12 users were abnormal users. A total of 120 data were collected from the normal user, and a total of 60 data were collected from the abnormal users, i.e., five data for each. In an attempt to increase the classification performance, the first 10 data of the normal user were considered to have been acquired during the adaptation period of the unique keypad, and thus discarded. Therefore, the total number of normal user data used for the tests was 110. Table 1 shows the data collection method, the number of participants, and the elements of the collected data.

Time (DT, FT1, FT2, FT3, FT4), pressure (downPressure, upPressure), and coordinate (downX, downY, upX, upY) were used as the data features. Table 2 presents the 62 touch data features used in the present study.

Given that the motion data were continuously collected until the user finished entering the PIN, the number of motion data for each data feature was not constant. The varying number of data points for each data feature made it difficult to compare overall results. The mean, root mean square (rms), sum, sum of positive numbers (pos), sum of negative numbers (neg), and standard deviation (std) of each vector’s axis data values were calculated and applied, and in this way suitable data feature values were extracted for the normalization of the motion data points varying in number. Six equations, shown below as Equations (3)–(8), were applied to the motion data. These six equations were applied to the data obtained for each of the *x*-, *y*-, and *z*-axes for each of the collected data features, including the acc, lacc, gyr, rot, and grot. As a result, a total of 18 data features were extracted. Table 3 shows the 90 motion data features collected by applying the six equations above.

- mean
  (3) $$S_{\left(mean\right)}=\frac{s_{1}+s_{2}+\cdots +s_{n}}{n}$$
- rms
  (4) $$S_{\left(rms\right)}=\sqrt{\frac{s_{1}^{2}+s_{2}^{2}+\cdots +s_{n}^{2}}{n}}$$
- sum
  (5) $$S_{\left(sum\right)}=\sum_{i=1}^{n}s_{i}$$
- pos
  (6) $$S_{\left(pos\right)}=\sum_{i=1}^{n}s_{i}(\mathrm{where}s_{i}>0)$$
- neg
  (7) $$S_{\left(neg\right)}=\sum_{i=1}^{n}s_{i}(\mathrm{where}s_{i}<0)$$
- std
  (8) $$S_{\left(std\right)}=\sqrt{\frac{\sum_{i=1}^{n}{\left(s_{i}-\bar{S}\right)}^{2}}{n}}(\mathrm{where}\bar{S}\mathrm{is}\mathrm{mean}\mathrm{of}S)$$

#### 5.1.2. Feature Selection

In the present study, the same feature selection method used in [4] was employed to improve classification performance, based on the extracted data. More specifically, the data features were filtered according to the calculated feature ranking: first, those in the lower 10% group were filtered, and then those in the lower 20% group were filtered. In similar fashion, the ratio went up to 50% in 10% increments. The collected data were divided into training data and test data at a ratio of 8:2. Here, the training data were used for feature selection and scaling. Classification test results obtained from these data were used to estimate the EER.

Feature selection is a process of selecting a set of sub-features related to the collected data, which is used to implement machine-learning classification. If there are too many data features, overfitting may occur. Thus, it is necessary to select some features that may be helpful to improve the ability of the system to classify normal and abnormal users who attempt authentication. In the present study, the feature scoring method used in [4] was selected as a feature selection method. The 152 features collected in the present study were ranked according to the values obtained using the feature scoring method proposed in [4]. The feature scoring equation in [4] can be expressed as Equation (9), which corresponds to the difference between the variation coefficient of the abnormal user data ($i\_cv(f_{i})$) and the variation coefficient of the normal user data ($u\_cv(f_{i})$) divided by the variation coefficient of the normal user data ($u\_cv(f_{i})$).

In this method, one reference value was set as the variation coefficient to compare the scores of features that have different values. The variation coefficient was calculated by dividing the standard deviation of the data by their mean value. By using this coefficient, it was possible to compare two or more data groups when their mean values were different from each other. In calculating the variation coefficient with the calculation method described in [4], the trimmed mean was used to reduce the effect of the standard deviation and outliers, as proposed in [4].

(9) $$feature\_score\left(f_{i}\right)=\frac{i\_cv\left(f_{i}\right)-u\_cv\left(f_{i}\right)}{u\_cv(f_{i})}$$

#### 5.1.3. Normalization

When data were collected by various sensors, there was a possibility that each data type may have a different unit, and thus they should be weighted accordingly. To prevent this, a normalization process was required. For data normalization, a standard scaling technique was used. Standard scaling refers to a method of converting the given data into one whose mean and standard deviation were 0 and 1, respectively. This data scaling was conducted using the following Equation (10). In Equation (10), $x_{i}$ denotes each datum of the data set *X*, $\bar{X}$ denotes a mean of the *X*, and std(X) denotes a standard deviation of the *X*.

(10) $$standardized\_value=\frac{x_{i}-\bar{X}}{std(X)}$$

#### 5.1.4. Classifiers

Three classifiers were applied to determine whether it was possible to classify normal and abnormal users based on the data collected using the unique keypad. The Linear SVM, One-Class SVM, and distance-based classifiers were used as classifiers for the tests.

- Linear SVM
  The linear SVM is designed to learn both normal and abnormal user data to create a linear decision boundary that can best classify normal and abnormal users. Once pre-separated data are entered, the linear SVM classifies the data to determine which user it would correspond to. This decision boundary is determined by the normalization parameters. Cost(c), a parameter that adjusts the overfitting during learning, is set to the default (1.0) value.
- One-Class SVM
  Unlike the linear SVM, the One-Class SVM learns only the normal user data and creates a suitable decision boundary for it. When pre-separated data are entered, the One-Class SVM classifies the data as corresponding to the normal user or not. In the tests, the One-Class SVM was trained with only the normal user data, and then used to classify abnormal user data. The parameter nu and gamma values used in this classifier can be changed. The nu, which represents the regression analysis precision, is set considering the misclassification cost range. The gamma is used to adjust the shape of the decision boundary curve that has been determined by the nu. The nu and gamma values were set to default (1.0) and 0.005, respectively.
- Distance-based Classifier
  The distance-based classifier is designed to measure the distance of each data with its own features for user classification. This classifier is sensitive when judging anomaly values, and data normalization is an important factor when it is applied. In the present study, only the normal user data were used to determine the threshold for the distance between the mean vector value of the normal user data and the training data. Any data exceeding the threshold were classified as corresponding to the abnormal users. The threshold was set according to the method presented in [4]. The distance between data points was calculated using the Manhattan distance metric. The Manhattan distance metric is used to determine the shortest distance between two points in *n*-dimensions as shown in Equations (11) and (12). The Manhattan distance is calculated using the following Equation (13).
  (11) $$X=\left\{x_{1},x_{2},\cdots ,x_{n}\right\}$$
  (12) $$Y=\left\{y_{1},y_{2},\cdots ,y_{n}\right\}$$
  (13) $$\begin{array}{cc} dist(X,Y) & =|x_{1}-y_{1}\left|+\right|x_{2}-y_{2}\left|+\cdots +\right|x_{n}-y_{n}|\\ \; & =\sum_{i=1}^{n}\left|x_{i}-y_{i}\right| \end{array}$$
  The Manhattan distance between each normal user data point and the mean value of the normal user data points was calculated. In order to allow most data (i.e., about 95% data) of the normal user to be classified as data from the normal user, the sum of the mean of the calculated values above, mean_distance, and their standard deviation, std_distance, multiplied by 2 was set as the threshold. The threshold value was calculated using the following Equation (14).
  (14) threshold=mean_distance+(std_distance×2)

### 5.2. Experimental Results

**Figure 5:**
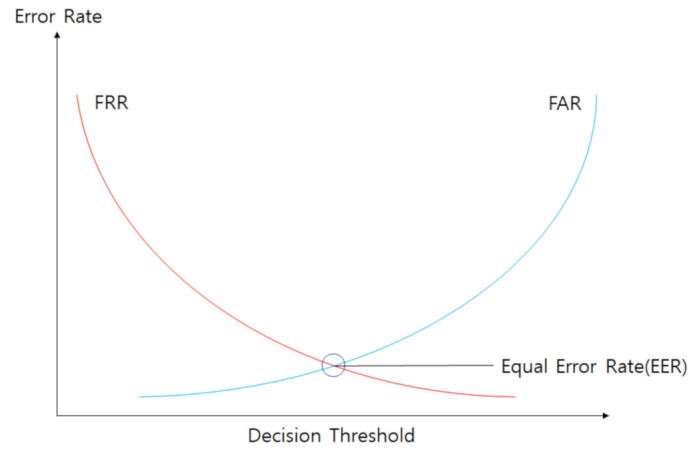
Equal Error Rate (EER).

The test results were compared based on the classification performance of the data collected using the unique keypad, as well as the results reported in [4]. A Manhattan distance-based classifier was implemented to have the same conditions as the one used in [4] so as to compare the results of the present study with those reported in [4]. The Linear SVM and the One-Class SVM were also applied to compare the data classification results obtained using the unique keypad. The results were compared by calculating the EER, an index that evaluates the data classification performance. An EER is a point where the FAR and FRR intersects. The FRR is the possibility that the system incorrectly classifies the normal user as being abnormal, whereas the FAR is the possibility that the system incorrectly classifies an abnormal user as being normal. The EER can be calculated using Equation (15). Figure 5 illustrates the concept of EER, which represents a point where the FAR and FRR become equal.

(15) $$EER=\frac{FRR+FAR}{2}$$

#### 5.2.1. Classification Results for Removing the Lower 10 % Features

**Figure 6:**
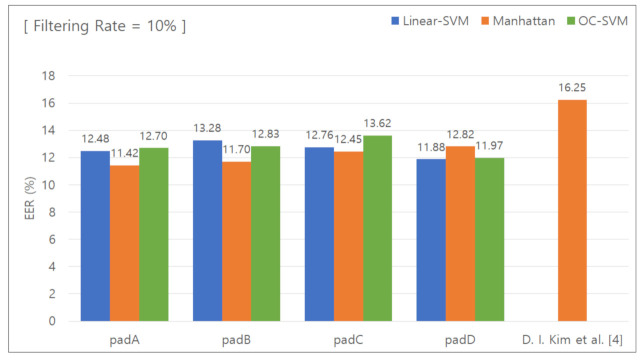
EER for removal of the lower 10% features.

Figure 6 shows the graph used to calculate the EER with the data features in the lower 10% group removed. Table 4 summarizes the calculated EER values. First, the results obtained using the Manhattan distance-based classifier were examined to compare the EER values of the unique keypad-based collected data and the conventional keypad-based collected data. When the unique keypad was used, the EER was 11.42%, 11.70%, 12.45%, and 12.82% for padA, padB, padC, and padD, respectively. When the conventional keypad was used, the EER was 16.25%. The difference in the EER was 4.83%, 4.55%, 3.8%, and 3.43%, respectively, and the average difference was 4.15%.

#### 5.2.2. Classification Results for Removing the Lower 20 % Features

**Figure 7:**
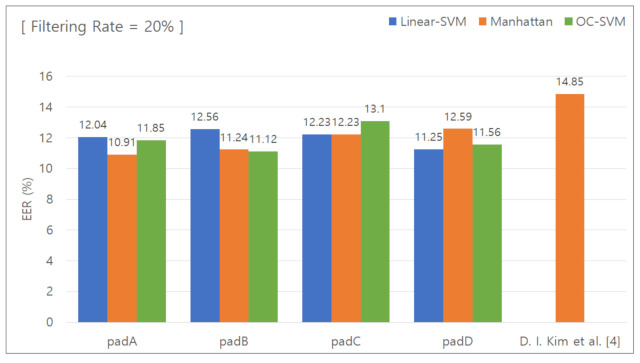
EER for removal of the lower 20% features.

Figure 7 presents the graph used to calculate the EER with the data features in the lower 20% group removed. Table 5 summarizes the calculated EER values. In the same way as above, the Manhattan distance-based classifier was used for comparison. When the unique keypad was used, the EER was 10.91%, 11.24%, 12.23%, and 12.59% for padA, padB, padC, and padD, respectively. When the conventional keypad was used, the EER was 14.85%. The difference in the EER was 3.94%, 3.61%, 2.62%, and 2.26%, respectively, and the average difference was 3.1%.

#### 5.2.3. Classification Results for Removing the Lower 30 % Features

**Figure 8:**
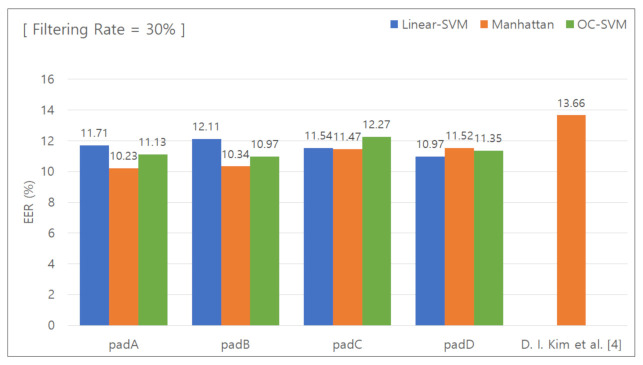
EER for removal of the lower 30% features.

Figure 8 presents the graph used to calculate the EER with the data features in the lower 30% group removed. Table 6 summarizes the calculated EER values. Additionally, the Manhattan distance-based classifier was used for comparison. When the unique keypad was used, the EER was 10.23%, 10.34%, 11.47%, and 11.52% for padA, padB, padC, and padD, respectively. When the conventional keypad was used, the EER was 13.66%. The difference in the EER was 3.43%, 3.32%, 2.19%, and 3.14%, respectively, and the average difference was 3.01%.

#### 5.2.4. Classification Results for Removing the Lower 40 % Features

**Figure 9:**
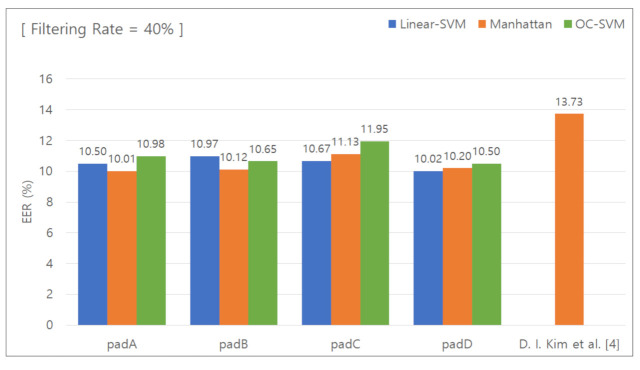
EER for removal of the lower 40% features.

Figure 9 presents the graph used to calculate the EER with the data features in the lower 40% group removed. Table 7 summarizes the calculated EER values. When the unique keypad was used, the EER was 10.01%, 10.12%, 11.13%, and 10.20% for padA, padB, padC, and padD, respectively. When the conventional keypad was used, the EER was 13.73%. The difference in the EER was 3.72%, 3.61%, 2.6%, and 3.53%, respectively, and the average difference was 3.36%.

#### 5.2.5. Classification Results for Removing the Lower 50 % Features

**Figure 10:**
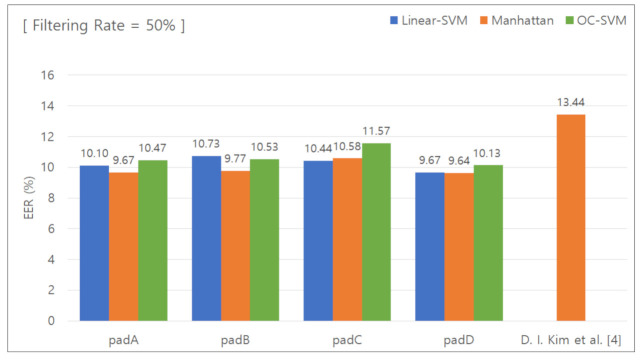
EER for removal of the lower 50% features.

Figure 10 presents the graph used to calculate the EER with the data features in the lower 50% group removed. Table 8 summarizes the calculated EER values. When the unique keypad was used, the EER was 9.67%, 9.77%, 10.58%, and 9.64% for padA, padB, padC, and padD, respectively. When the conventional keypad was used, the EER was 13.44%. The difference in the EER was 3.77%, 3.67%, 2.86%, and 3.8%, respectively, and the average difference was 3.52%.

#### 5.2.6. Analysis of the Experimental Results

The classification performance results obtained using the unique keypad were compared with those obtained using a conventional keypad in [4] to determine the effect of the keypad type on the classification performance. When the Manhattan distance-based classifier was used, the EER was 11.42%, 11.70%, 12.45%, and 12.82% for padA, padB, padC, and padD, respectively, with the data features in the lower 10% group removed. When compared to 16.25% reported in [4], the EER was 3.43 to 4.82% lower. It was found that, as the filtering process proceeded further, the measured EER continued to decrease. With the data features in the lower 50% group removed, the EER was 9.67%, 9.77%, 10.58%, and 9.64%. When the data features in the lower 50% group were removed, the EER was 13.44% in [4]. The difference in the EER ranged from 2.86% to 3.8%. These results indicated that the classification performance increased with increasing feature selection ratio. This also showed that the unique keypad-based collected data contributed to improving the ability of the system to classify the normal user.

The user classification performance of the four unique keypads was compared to determine the optimum classifier for the keypads. The tests were conducted while varying the feature removal ratio from the lower 10% to the lower 50%. The results showed that the best performance was achieved when the data features in the lower 50% group were removed. When the data features in the lower 50% group were removed, which provided the best performance, the EER was the lowest when the Manhattan distance-based classifier was used among the three classifiers (the Linear SVM, Manhattan distance-based classifier, and One-Class SVM) at 9.67%, 9.77%, and 9.64% for padA, padB, and padD, respectively. padC exhibited the lowest EER at 10.44% when the Linear SVM was employed. These results confirmed that the Manhattan distance-based classifier, which classifies subjects based on a threshold, was the optimal option for user classification.

## 6. Conclusions

In the present study, a unique keypad assigned only to the normal user was proposed to improve user classification performance for a keystroke dynamics-based smartphone user authentication process. Based on the Mersenne twister algorithm, randomly generated numbers were used to form a keypad that is familiar only to the normal user. User classification tests were conducted using the proposed unique keypad to confirm that the unique keypad exhibited the uniqueness and consistency that represented the normal user only, while providing better user classification performance than conventional keypads.

The data collected using the unique keypad were compared with those collected using a conventional keypad to compare their data classification performance. Additionally, three classifiers were tested using the unique keypad-based collected data to determine their user classification performance. For the proper comparison of classification performance with conventional keypads, the data features collected through the smartphone and feature selection methods were all set the same. As a result, it was found that the classification performance was better when the unique, rather than the conventional keypad, was used. Additionally, four different unique keypads exhibited the best data classification performance when the Manhattan distance-based classifier was employed. This confirmed that the Manhattan distance-based classifier was the most suitable for data classification. Based on the major findings of the present study, the data collection method using the unique keypad proposed in this study has a high potential for application as one of the data collection methods for keystroke data-based authentication systems.

Based on the results of the present study, our future study will focus on developing the different data features of keypads, such as the button size, character addition, efficient arrangement, and user classification. This will help generate a unique keypad for every user, which will contribute to improving the user classification performance, because it will collect features that can only be collected from the normal user. Even if a smartphone user’s PIN, as the ultimate password, has been hacked, there will be no need to worry because this technology will help protect their private information in an even safer way.

## Figures and Tables

**Table 1:** Collection data element.

Data Collection	
Method	Nexus 5X Device with an Android Application
Subject	1 Legitimate User and 12 Impostors per Smartphone
Element	Touch Sensor - Time, Pressure, Coordinate Accelerometer Sensor—Acc Gyro Sensor—Gyr Software-based Sensor Value—Lacc, Rot, Grot

**Table 2:** The number of touch data features.

Time					Pressure		Coordinate			
DT	FT1	FT2	FT3	FT4	downPressure	upPressure	downX	downY	upX	upY
6	5	5	5	5	6	6	6	6	6	6
62 Touch Data Features										

**Table 3:** The number of motion data features.

6 Features per Each Axis of *x*, *y*, *z* (mean, rms, sum, pos. neg, std)				
acc	lacc	gyr	rot	grot
18	18	18	18	18
90 Motion Data Features				

**Table 4:** EER for removal of the lower 10% features.

Pad	Linear SVM	Manhattan Distance-Based Classifier	One-Class SVM
PadA	12.48%	11.42%	12.70%
PadB	13.28%	11.70%	12.83%
PadC	12.76%	12.45%	13.62%
PadD	11.88%	12.82%	11.97%

**Table 5:** EER for removal of the lower 20% features.

Pad	Linear SVM	Manhattan Distance-Based Classifier	One-Class SVM
PadA	12.04%	10.91%	11.85%
PadB	12.56%	11.24%	11.12%
PadC	12.23%	12.23%	13.10%
PadD	11.25%	12.59%	11.56%

**Table 6:** EER for removal of the lower 30% features.

Pad	Linear SVM	Manhattan Distance-Based Classifier	One-Class SVM
PadA	11.71%	10.23%	11.13%
PadB	12.11%	10.34%	10.97%
PadC	11.54%	11.47%	12.27%
PadD	10.97%	11.52%	11.35%

**Table 7:** EER for removal of the lower 40% features.

Pad	Linear SVM	Manhattan Distance-Based Classifier	One-Class SVM
PadA	10.50%	10.01%	10.98%
PadB	10.97%	10.12%	10.65%
PadC	10.67%	11.13%	11.95%
PadD	10.02%	10.20%	10.50%

**Table 8:** EER for removal of the lower 50% features.

Pad	Linear SVM	Manhattan Distance-Based Classifier	One-Class SVM
PadA	10.10%	9.67%	10.47%
PadB	10.73%	9.77%	10.53%
PadC	10.44%	10.58%	11.57%
PadD	9.67%	9.64%	10.13%
